# Complications and prognosis of patients undergoing apical or septal right ventricular pacing

**DOI:** 10.1136/openhrt-2018-000962

**Published:** 2019-02-09

**Authors:** Nick B Spath, Kelvin Wang, Sowmya Venkatasumbramanian, Omar Fersia, David E Newby, Chris CE Lang, Neil R Grubb, Marc R Dweck

**Affiliations:** 1 BHF/University Centre for Cardiovascular Science, University of Edinburgh, Edinburgh, UK; 2 Edinburgh Heart Centre, Royal Infirmary of Edinburgh, Edinburgh, UK

**Keywords:** right ventricular pacing, septal pacing, apical pacing

## Abstract

**Objectives:**

Optimal right ventricular lead placement remains controversial. Large studies investigating the safety and long-term prognosis of apical and septal right ventricular lead placement have been lacking.

**Methods:**

Consecutive patients undergoing pacemaker insertion for high-degree atrioventricular block at Edinburgh Heart Centre were investigated. Periprocedural 30-day complications were defined (infection/bleeding/pneumothorax/tamponade/lead displacement). Long-term clinical outcomes were obtained from the General Register of Scotland and electronic medical records. The primary endpoint was a composite of all-cause mortality, new heart failure, hospitalisation for a major cardiovascular event, as per the CArdiac REsynchronization in Heart Failure trial. Secondary endpoints were all-cause mortality, new heart failure and their composite.

**Results:**

820 patients were included, 204 (25%) paced from the septum and 616 (75%) from the apex. All baseline variables were similar with the exception of age (septal: 73.2±1.1 vs apical: 76.9±0.5 years, p<0.001). Procedure duration (58±23 vs 55±25 min, p=0.3), complication rates (18 (8.8) vs 46 (7.5)%, p=0.5) and postimplant QRS duration (152 (23) vs 154 (27) ms, p=0.4) were similar. After 1041 days (IQR 564), 278 patients met the primary endpoint, with no difference between the septal and apical groups in unadjusted (HR 0.86 (95% CIs 0.64 to 1.15)) or multivariable analysis correcting for age, gender and comorbidity (HR 0.97 (95% CI 0.72 to 1.30)). Similarly, no differences were observed in the secondary endpoints.

**Conclusions:**

This large real-world cohort of patients undergoing right ventricular lead placement in the septum or apex demonstrated no difference in procedural complications nor long-term clinical outcomes. Both pacing strategies appear reasonable in routine practice.

**Key questions**

**What is already known about this subject?**

- There remains a lack of consensus on optimal right ventricular lead placement.

**What does this study add?**

- Periprocedural complication rates and long-term clinical outcomes for septal and apical right ventricular lead placement are similar.
- Fluoroscopy-guided right ventricular septal lead placement does not consistently result in a narrow paced QRS duration.
- Patients in whom narrow paced QRS duration is achieved have a favourable long-term clinical outcome.

**How might this impact on clinical practice?**

- Reduction of QRS duration is crucial and should be prioritised when placing right ventricular leads.
- Pacing strategies for routine clinical practice that result in more consistent reduction of QRS should be the focus of future research in this field.

## Introduction

Cardiac pacing devices are central to modern cardiology with over 500 000 devices implanted in the annually across Europe.^1^ Higher degree atrioventricular block remains an important indication for pacing^2^ in order to mitigate against the risk of syncope, progressive heart failure and sudden cardiac death. However, there remains a lack of consensus on the optimal positioning of the right ventricular lead in terms of cardiac function and long-term clinical outcomes.^3^

Conventionally, right ventricular leads are placed at the apex, but increasing evidence suggests this strategy may have deleterious effects on cardiac function by producing an iatrogenic left bundle branch block (LBBB) pattern on the ECG and dyssynchronous ventricular contraction.^2 4^ The LBBB ECG pattern is associated with worse clinical outcomes in both diseased and normal hearts,^5–8^ with recent data suggesting that pacemaker-related LBBB is similarly disadvantageous. Indeed patients with severely impaired left ventricular function and high right ventricular apical pacing burdens (50%–100%) have an increased subsequent incidence of heart failure compared with patients with low burdens (0%–50%).^9^ This is thought to relate to the interventricular and intraventricular electrical and mechanical dyssynchrony^2 7 10^ that occurs with apical pacing, which can lead to adverse remodelling,^11^ altered cardiac perfusion^12^ and impaired function.^13^ Even in patients with preserved left ventricular systolic function, there is evidence to suggest some reduction in function with both apical and septal pacing.^14^

Alternative pacing strategies achieving more physiological depolarisation might improve ventricular synchrony and protect against these detrimental effects. These include minimal ventricular pacing algorithms, upgrade to cardiac resynchronisation therapy and His-bundle pacing.^15^ However, the most widely used strategy is pacing of the right ventricular septum and outflow tract.^2 7^ The rationale is that pacing from these septal sites might allow recruitment of the intrinsic cardiac conduction system that lies in close proximity, thereby reducing QRS duration and subsequent ventricular dyssynchrony.^15^ Septal pacing is also attractive because it is less technically challenging than other strategies such as cardiac resynchronisation therapy and His-bundle pacing.^14 15^ Moreover, it is generally accepted that septal lead placement avoids the perioperative risk of cardiac perforation and tamponade compared with apical lead placement. However, concerns have been raised about the risks of lead displacement and the ability of this approach to reliably recruit the intrinsic conduction system.^15 16^

In the present study, we aimed to investigate the procedural safety and long-term clinical outcomes of a large real-world cohort of patients with higher degree atrioventricular block non-selectively assigned to pacing operators with preference for either septal or apical right ventricular pacing strategies.

## Methods

**Figure 1:**
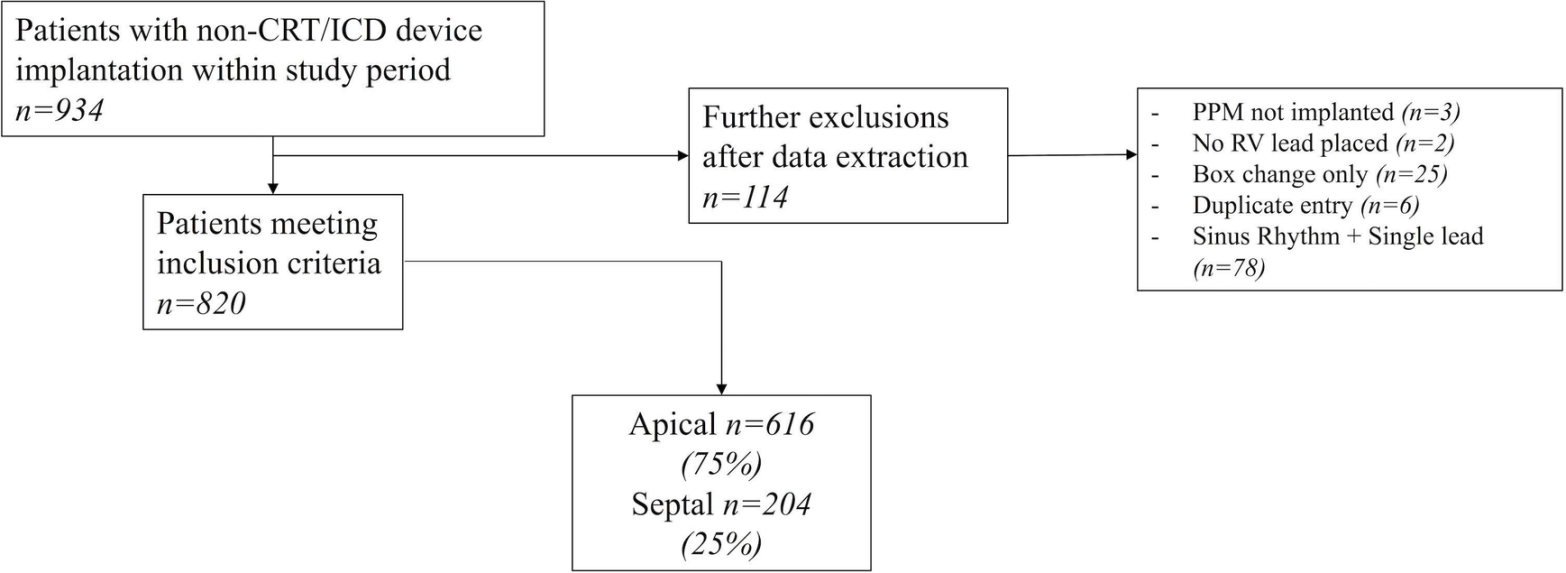
Study flow chart, with exclusions. CRT, Cardiac Resynchronisation Therapy; ICD, Impantable Cardioverter Defibrillator; PPM, Permanent Pacemaker; RV, Right Ventricle.

Consecutive patients undergoing pacemaker device implantation from 16 April 2010 to 29 September 2016 at the Edinburgh Heart Centre were included in the study. Over the study period, there were five operators with two favouring septal right ventricular lead placement and three favouring apical lead placement. All septal lead placements were achieved with active fixation leads using stylets fashioned by the operators to facilitate septal positioning. No preshaped stylets or steerable sheaths were used. The final septal positions were confirmed using fluoroscopy (posteroarterior (PA) and left anterior oblique (LAO) 40 projections) and assessment of the current of injury. In comparison, most apical lead placements were achieved with active fixation leads (n=483, 78.4%) using PA and right anterior oblique fluoroscopy projections. Patients listed for permanent pacemakers were placed in a central pool and then non-selectively allocated to 1 of 6 weekly lists at our institution. Inclusion criteria were defined to select patients undergoing new right ventricular lead implantation (either new device implants or ventricular lead repositioning) for high-degree atrioventricular block (second/Mobitz type 2 or third-degree atrioventricular block). Exclusion criteria were pacemaker implantation for isolated sick sinus syndrome or other conditions associated with low ventricular pacing burdens and patients undergoing cardiac defibrillator or resynchronisation therapy system implantation. Patients in sinus rhythm undergoing single-lead device implantation were excluded in an attempt to exclude frail and unstable patients with an inherently poor prognosis who may be more likely to have an apical lead placed (figure 1). Clinical audit approval for this study was provided by the Edinburgh Heart Centre.

### Data collection

Data were collected using a prespecified collection protocol and standardised case report form from electronic medical records. Baseline clinical and procedural data were collected by a single observer (KW) blinded to clinical outcomes. Data on preimplantation medication and comorbid conditions were recorded including atrial arrhythmias, congestive heart failure (symptomatic or echocardiographic ejection fraction <50%), chronic kidney disease (estimated glomerular filtration rate <60 mL/min/1.73 m^2^), liver failure, metastatic cancer, stroke or transient ischaemic attack, diabetes mellitus, hypertension (persistent blood pressure 140/90 mm Hg or higher), ischaemic heart disease (symptomatic of angina pectoris or imaging diagnosis), previous coronary arterial bypass grafting, valvular heart disease (murmur or echocardiographic diagnosis), previous valvular surgery and interventricular conduction delays (table 1). Procedural information was collected (KW) from electronic records and a national audit tool for cardiac procedures (St. Thomas’ Cardiac Audit Tool, TOMCAT) that routinely captures screening times, procedure times (patient time in theatre) and procedural complications. Procedural complications were prespecified as bleeding/haematoma, pneumothorax, lead displacement, infection, cardiac tamponade, atrial lead dysfunction, right ventricular lead repositioning, right ventricular perforation without tamponade, wound dehiscence, pericarditis and subclavian vein thrombosis (table 2). A composite procedural complication score was calculated as the sum of each of these complications for patients undergoing apical and septal lead placement. The final right ventricular lead position was determined by the primary operator, using fluoroscopy and documented in the electronic medical records. Where available, postimplantation ECGs were assessed, and the duration of the paced QRS complexes recorded.

**Table 1:** Demographic comparison of apical and septal groups (n (%) or mean±SD)

	Apical (n=616)	Septal (n=204)	P value
Age (year)	77±11	73±15	**<** **0.001**
Gender (♂)	382 (62)	125 (61)	>0.9
Urgency (urgent)	396 (65)	136 (67)	0.7
Dual/single chamber (dual)	565 (92)	193 (95)	0.2
Comorbidities			
Atrial arrhythmia	152 (25)	41 (21)	0.3
Heart failure	55 (9)	17 (9)	>0.9
Renal failure	87 (14)	26 (13)	0.8
Liver failure	0	0	
Metastatic cancer	7 (1)	0 (0)	0.3
Stroke	86 (14)	22 (11)	0.3
Diabetes mellitus	122 (20)	37 (19)	0.8
Hypertension	416 (68)	123 (62)	0.1
Ischaemic heart disease	162 (27)	58 (29)	0.5
CABG	37 (6)	19 (10)	0.1
Valvular heart disease	172 (28)	62 (31)	0.5
Valve surgery	52 (9)	22 (11)	0.3
Haematology/biochemistry			
Haemoglobin	128±19	128±19	>0.9
Creatinine	98±55	99±77	0.7
Sodium	138±4	138±4	0.2
Medications			
Diuretic	205 (34)	61 (31)	0.5
ACEI/ARB	270 (44)	78 (39)	0.2
Beta-blocker	80 (13)	27 (14)	0.9
Digoxin	4 (1)	0 (0)	0.6
Ca-channel blocker	103 (17)	30 (15)	0.7
Amiodarone	9 (2)	4 (2)	0.8

ACEI, angiotensin converting enzyme inhibitor; ARB, angiotensin II receptor blocker; CABG, coronary artery bypass grafting; eGFR, estimated glomerular filtration rate.

**Table 2:** Thirty-day complication rates between apical and septal cohorts (n (%))

Complication at 30 days	Apical group (n=616)	Septal group (n=204)	P value
Bleeding/haematoma	8 (1)	6 (3)	0.1
Pneumothorax	16 (2)	5 (2)	>0.9
Lead displacement	6 (1)	3 (2)	0.6
Infection	9 (2)	3 (2)	>0.9
Cardiac tamponade	0 (0)	1 (1)	0.2
Other*	7 (1)	0 (0)	0.1
Composite periprocedural complication score	**46** (**8%**)	**18** (**9%**)	**0.5**

*Atrial lead dysfunction, right ventricular lead repositioning, right ventricular perforation without tamponade, wound dehiscence, pericarditis and subclavian vein thrombosis.

### Clinical outcomes

Clinical outcome data were collected from the General Register of Scotland and electronic medical records, by an observer blinded to the baseline demographic and procedural data (NS). Our composite primary endpoint was prespecified and based on the CArdiac REsynchronization in Heart Failure trial^17^: all-cause mortality, congestive heart failure and hospitalisation for a major cardiovascular event. This was considered an appropriate primary endpoint as the mechanism by which non-apical pacing is proposed to improve clinical outcomes is similar to that of cardiac resynchronisation therapy by reducing QRS duration. The earliest event was used for the time-to-endpoint analysis. Prespecified secondary endpoints were all cause mortality, new-onset congestive heart failure and the composite of the two. A post hoc analysis examined a composite secondary endpoint of all-cause mortality, congestive heart failure, myocardial infarction, unstable angina, stroke, cardiac arrest and arrhythmia. Finally, an outcome analysis based on the achieved paced QRS duration was also undertaken.

### Statistical analysis

Statistical analysis was carried out in R (V.2.15.2, Vienna, Austria) and GraphPad Prism (V.7.0, Graphpad Software Inc., La Jolla, California, USA). Continuous variables were assessed using parametric and non-parametric tests as appropriate, while categorical variables were compared using the Fisher’s exact test. Cox regression modelling was performed to define the HRs for all primary and secondary endpoints after adjusting for age, sex and comorbidities (chronic kidney disease, congestive heart failure, hypertension, diabetes mellitus and ischaemic heart disease). A post hoc Cox regression analysis with selected relevant clinical endpoints was also carried out. Finally, we performed a subgroup analysis to investigate the breadth of the paced QRS duration in patients with a septal lead (narrow complex paced QRS <130 ms vs broad complex paced QRS ≥130 ms) and the effect of paced QRS duration on the primary endpoint.

## Results

In total, 820 eligible patients were identified with 616 (75%) in the apical and 204 (25%) in the septal right ventricular pacing groups. These two groups were well balanced for all baseline clinical variables including gender, comorbidities, medication and routine baseline laboratory tests. The sole exception was age with patients having a septal lead being on average 3.7 years younger than those with apical leads (table 1).

**Figure 2:**
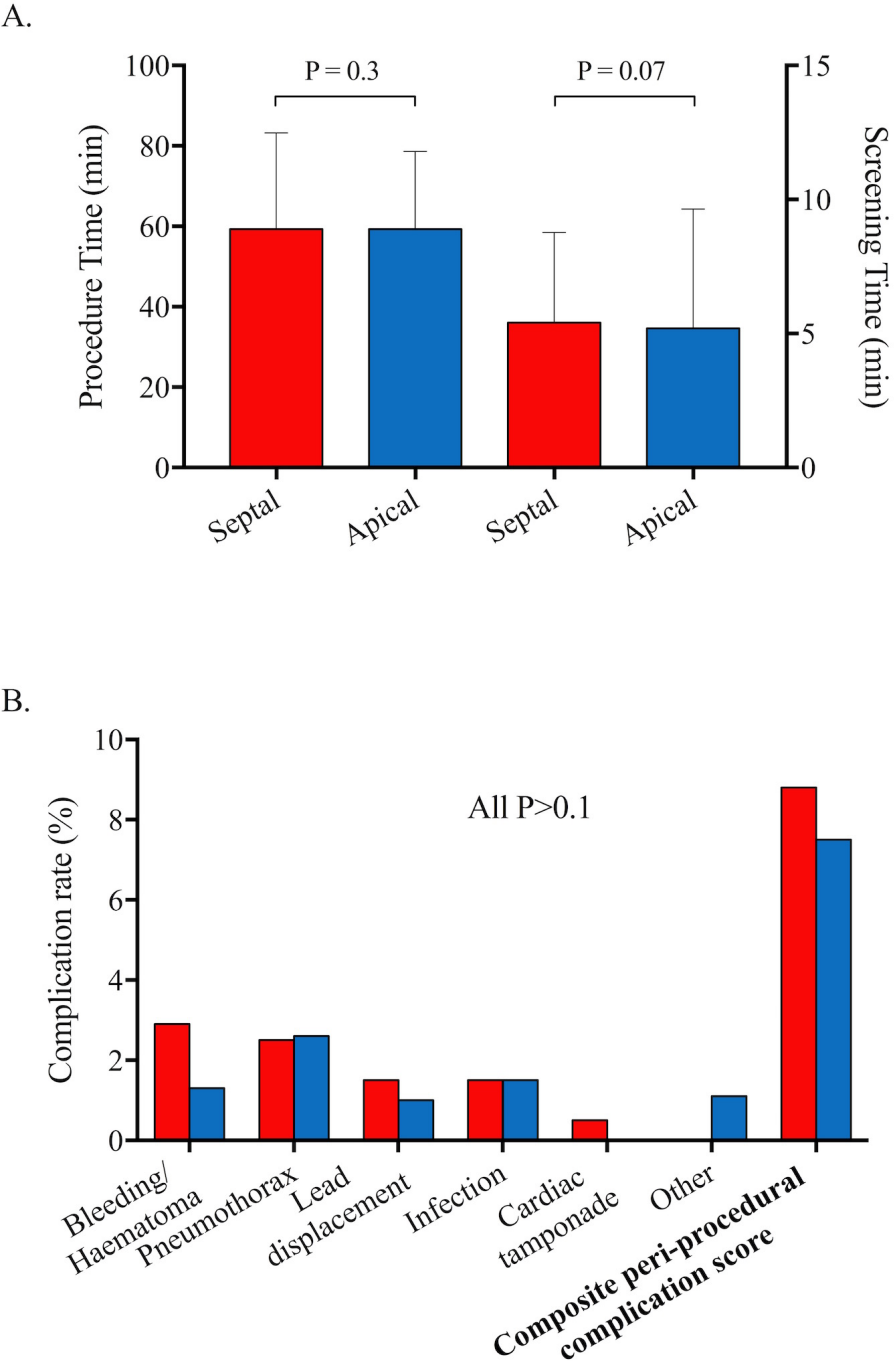
Mean procedure time and screening time (A, error bars indicate SD) and 30-day complication rates and (B) for septal (red) and apical (blue) patient groups.

There was no difference in procedure times (defined as time from skin preparation to skin closure) between apical and septal groups (58±23 vs 55±25 min, respectively, p=0.34) or fluoroscopic screening times (defined as total screening time per procedure, 4.0±3.9 vs 4.9±3.6 min, respectively, p=0.07). Pneumothorax, infection, bleeding and haematoma were the most frequent complications with no observed differences between the two groups in their incidence (table 2) or in the composite 30-day periprocedural complication score (7.5% (n=46) vs 8.8% (n=18), respectively, p=0.5, figure 2).

**Figure 3:**
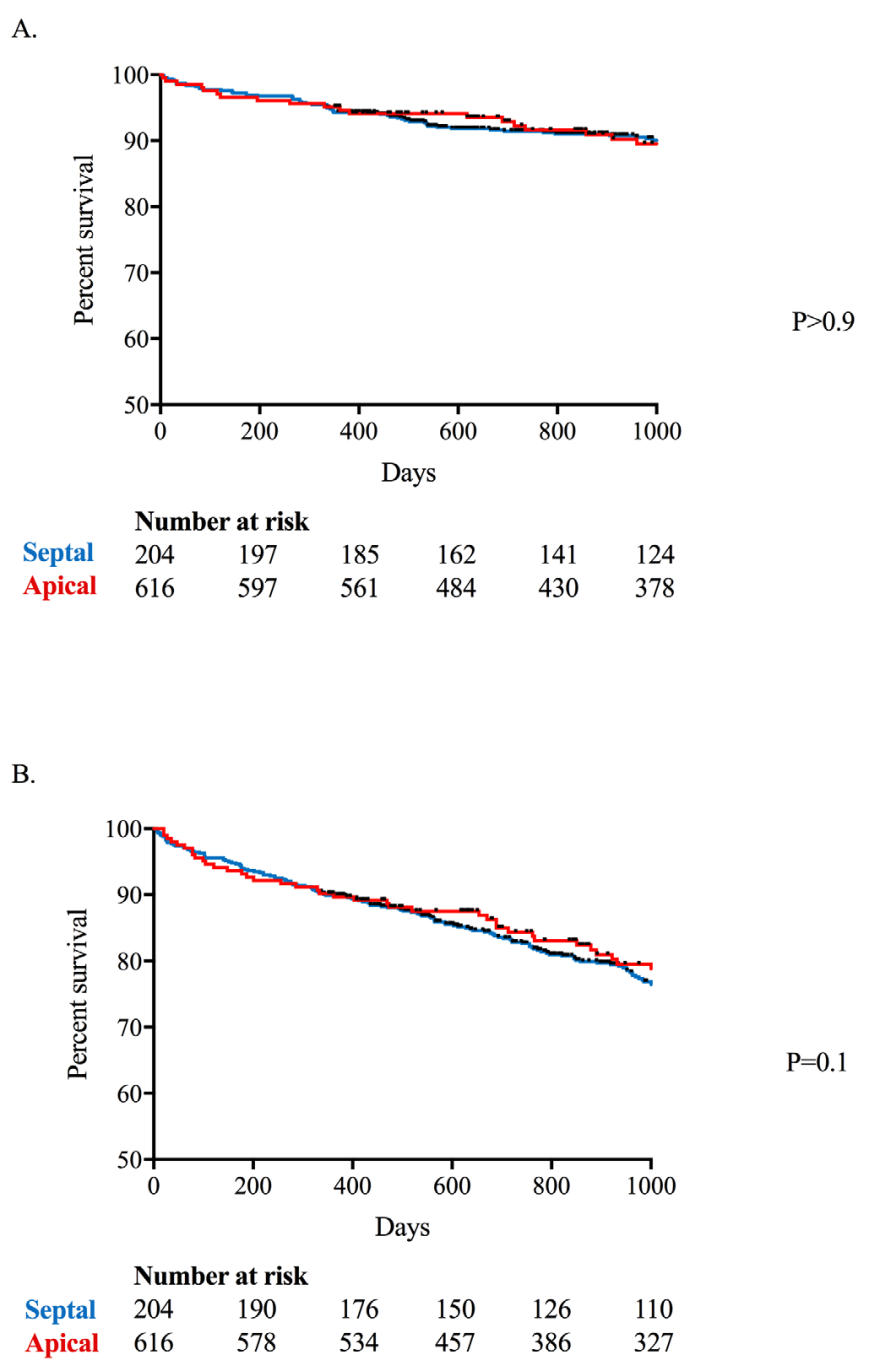
Kaplan-Meier plot for the prespecified primary endpoint (CARE-HF (A) and the secondary composite endpoint (all-cause mortality and new onset heart failure (B) in the septal (red) and apical (blue) cohorts. Median follow-up of 1000 days. CARE-HF, CArdiac REsynchronization in Heart Failure trial.

After a median of 1041 (IQR 564) days follow-up, 278 patients met the primary endpoint (table 3). There was no difference in the primary endpoint between the septal and apical pacing groups in either unadjusted (HR 0.86, 95% CIs 0.64 to 1.15) or multivariable analyses after correcting for age and gender (HR 1.02, 95% CIs 0.76 to 1.36), and age, gender and presence of comorbidities (chronic kidney disease/congestive heart failure/hypertension/diabetes mellitus/ischaemic heart disease, HR 0.97, 95% CIs 0.72 to 1.30). Similarly, no differences between groups were observed for any of the prespecified or post hoc secondary endpoints (table 4, figure 3). In the post hoc anlaysis where pulmonary embolism and ruptured aortic aneurysm were removed from the composite endpoint, there was no difference between septal or apical groups in either unadjusted (HR 0.91, 95% CIs 0.68 to 1.21) or multivariable analyses correcting for age and gender (HR 1.08, 95% CIs 0.81 to 1.45), and age, gender and comorbidity (HR 1.02, 95% CIs 0.76 to 1.38).

**Table 3:** Clinical outcomes in patients undergoing apical and septal right ventricular pacemaker implantation (n (%))

Clinical endpoint	Apical group (n=616)	Septal group (n=204)	P value
All-cause mortality	181 (29)	47 (23)	0.1
Unstable angina	8 (1)	5 (3)	0.3
Myocardial infarction	38 (6)	16 (8)	0.4
Pulmonary embolism	7 (1)	1 (1)	0.7
Cardiac arrest	6 (1)	1 (1)	>0.9
Paroxysmal SVT/VT	41 (7)	4 (2)	0.01
Atrial fibrillation/flutter	43 (7)	14 (7)	>0.9
Heart failure	38 (6)	10 (5)	0.6
Stroke	15 (2)	8 (4)	0.3
Ruptured AAA	6 (1)	1 (1)	>0.9
Total	383 (62)	116 (57)	0.2

AAA, abdominal aortic aneurysm; SVT, supraventricular tachycardia; VT, ventricular tachycardia.

**Table 4:** Cox regression modelling; HRs for septal pacing for primary and secondary endpoints

Endpoint	Model	HR	95% CIs
Primary composite endpoint	1 (Unadjusted)	0.86	0.64 to 1.15
	2 (Age, gender)	1.02	0.76 to 1.36
	3 (Age, gender comorbidities*)	0.97	0.72 to 1.30
Secondary endpoints			
All-cause mortality	3 (Age, gender comorbidities*)	0.92	0.66 to 1.28
New-onset heart failure	3 (Age, gender comorbidities*)	0.69	0.24 to 1.67
All-cause mortality/new onset heart failure†	3 (Age, gender comorbidities*)	0.90	0.65 to 1.23
Modified composite endpoint‡	1 (Unadjusted)	0.91	0.68 to 1.21
	2 (Age, gender)	1.08	0.81 to 1.45
	3 (Age, gender comorbidities*)	1.02	0.76 to 1.38

*Chronic kidney disease/congestive heart failure/hypertension/diabetes mellitus/ischaemic heart disease.

†All-cause mortality/new-onset heart failure.

‡As per CARE-HF, with pulmonary embolism and ruptured aortic aneurysm excluded.

CARE-HF, CArdiac REsynchronization in Heart Failure trial.

**Figure 4:**
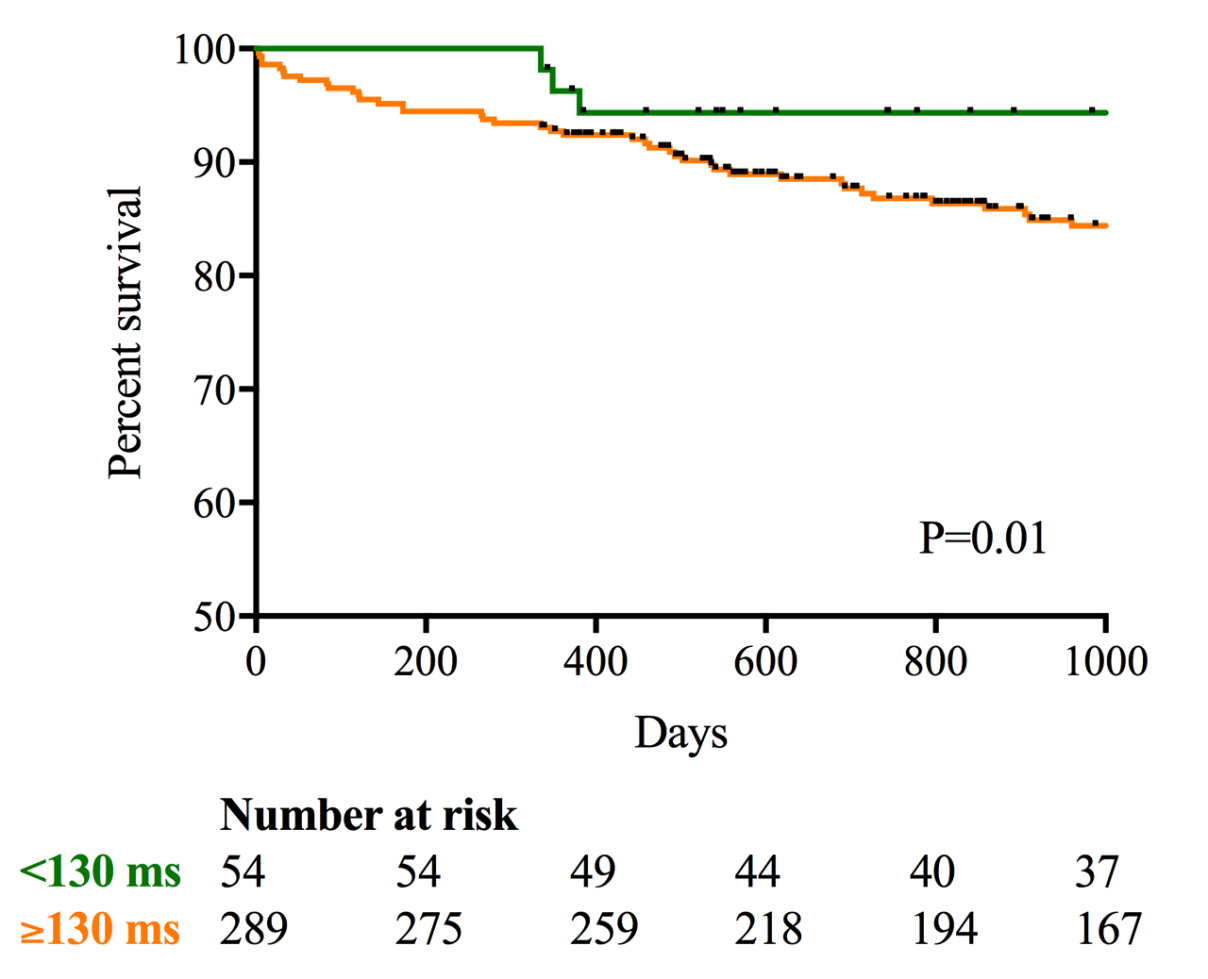
Kaplan-Meier plot for the primary composite endpoint in patient with a narrow paced QRS (<130 ms; green) versus broad paced QRS duration (≥130 ms; orange). Median follow-up of 1000 days.

In a subgroup (n=343) where paced postimplant electrocardiograms were available, there were no differences in paced QRS duration between the septal and apical groups (mean 152±23 (n=87) ms vs 154±27 (n=256) ms, respectively, p=0.4). Across the cohort as a whole, 16% of patients (n=14, 6.9% septal implants; n=40, 6.5% apical implants, p=0.9) demonstrated a narrow paced QRS (<130 ms) (excluding fusion and pseudofusion beats), while 84% (n=289) had a broad paced QRS (≥130 ms). Interestingly, patients in whom a narrow paced QRS (<130 ms) was achieved, irrespective of pacing site, demonstrated improved clinical outcomes compared with patients with a broad QRS (primary composite endpoint; 6% vs 17%, p=0.04). Kaplan-Meier curve analysis confirmed this finding with improved time-to-event outcomes in the narrow complex group (p=0.01; figure 4).

## Discussion

In this large-scale retrospective analysis, where patients underwent right ventricular septal or apical pacing, we have demonstrated that right ventricular septal pacing is a safe alternative to the conventional apical approach with no increase in periprocedural complication rates or procedural times. However, septal pacing did not result in improved long-term clinical outcomes, perhaps because it did not consistently result in a narrow complex paced QRS. Both right ventricular septal and apical pacing are therefore reasonable strategies with further work required to develop simple approaches that more consistently reduce the paced duration of the QRS.

While not a prospective randomised trial, the present study provides analysis of real-world consecutive patient data at a tertiary referral centre, where patients are non-selectively assigned from a central pool to pacing lists performed by operators with a preference for either apical of septal lead placement. As a consequence, the right ventricular septal and apical pacing cohorts were well balanced across every baseline clinical characteristics that were collected with the sole exception of age. Interestingly, the septal cohort was on average 3.7 years younger, perhaps reflecting the tendency to be less aggressive in striving for a septal pacing site in elderly patients. We therefore provide a robust and large real-world dataset with which to investigate the procedural safety and long-term clinical outcomes of septal versus apical pacing.

Septal lead placement poses technical challenges,^16–18^ raising concerns about lead displacement and the increased risk of complications with longer procedures. However, in this present study, we observed no difference in procedure duration, fluoroscopy times and periprocedural complication rates between the apical and septal cohorts. This was true both when considering each of the complications individually as well as the composite risk score, confirming septal lead placement as a readily feasible and safe alternative to apical pacing. Indeed, complication rates observed across our cohort as a whole were comprabale with other reported studies.^19 20^

Our second aim was to investigate whether septal pacing is associated with a favourable long-term prognosis. Despite a high event rate, no difference was observed between the right ventricular septal and apical pacing groups in the primary composite clinical endpoint, at median follow-up of 1041 days. Similarly, no difference was observed for any of the secondary clinical endpoints in multivariable analysis. What is the explanation for this result given the well-established literature outlining the adverse outcomes associated with LBBB and right ventricular apical pacing? Selection bias has potential to favour apical lead placement given that operators may be more likely to attempt septal lead implantation in patients with known left ventricular impairment. However, the high degree of similarity in comorbidity and medication between our patient groups suggests this was not the case. It is the analysis of the postimplantation ECGs that appears to provide the answer. In this large subgroup (n=343), no difference was observed in the paced QRS duration between the septal and apical pacing groups. Indeed, only 16% of the septal group achieved a narrow paced QRS (<130 ms), similar to the proportion in the apical cohort. Septal pacing therefore failed to reduce ventricular dyssynchrony compared with apical pacing, thereby explaining the similar outcomes. One potential explanation for this failure is that fluoroscopy is insufficient to guide accurate positioning of the pacing lead on to the septum. Recent studies have confirmed that only a minority of right ventricular leads are placed in the true septum using conventional fluoroscopic views and some have advocated the use of individualised LAO projection and echocardiography to confirm septal lead positioning.^16 21^ Alternatively, it may reflect variations in the anatomy of the His-Purkinje system. High septal right ventricular outflow tachycardia produces a broad complex QRS. It is perhaps unsurprising therefore that pacing in this region fails to achieve a narrow paced QRS. In comparison, fascicular tachycardia arising from the distal fascicles of the left bundle branch may produce a relatively narrow complex tachycardia. Pacing in this region might similarly result in a narrow apical paced QRS, potentially explaining the patients with a narrow paced QRS in the apical group.

Interestingly, the patients in whom a narrow QRS paced rhythm (<130 ms) was achieved, irrespective of pacing site, did demonstrate a lower risk of future adverse events compared with those with a broad QRS≥130 ms (0.05% vs 18.8%, respectively, p=0.01). This is consistent with the hypothesis that pacemaker-related LBBB is associated with an adverse prognosis and supports strategies that aim to achieve narrow complex pacing. Successful reduction in QRS duration can be achieved with cardiac resynchronisation therapy, but this is time consuming. His bundle pacing is an alternative strategy with potential to produce the same QRS duration and mechanical function as atrial pacing.^22^ Technical challenges have also been reported with His bundle pacing including low success rates (65%), high complications rates, problems with ventricular sensing and reduced generator lifespan due to higher pacing thresholds.^23^ However, Abdelrahman and colleagues demonstrated encouraging results in a recent observational cohort study of 765 consecutive patients.^24^ While the average procedure and screening times were longer than for conventional pacing (70 and 10 mins vs 55 and 7 mins, respectively, p<0.001) and there was an increased rate of ventricular lead revision, the authors demonstrated a signficiant reduction in QRS duration with His bundle pacing (128 (SD 27.7) ms vs 166 (21.8) ms, respectively) and a reduction in the composite clinical endpoint at 5 years. Improved strategies for septal pacing should also be explored, for example, by using steerable introducer sheaths, patient-tailored fluoroscopic projections and using intraoperative QRS duration and using QRS duration rather than fluoroscopic appearances to better guide lead placement on to the septum.

### Limitations

Echocardiography and pacing burden data were not available for analysis. This study was a retrospective analysis at a single centre and so is inherently limited by unforeseen counfounding factors and lack of randomisation. However, the system of non-selective patient allocation to pacing operators resulted in the balancing of all clinical variables examined with the exception of age. Similar balancing might therefore be expected among the unknown confounders.

## Conclusion

In this large real-world observational analysis, procedural safety and long-term clinical outcomes were similar between patients undergoing apical and septal right ventricular pacing. While septal pacing appears a safe and feasible strategy, further work is required to develop methods that can more consistently deliver narrow QRS right ventricular pacing.
